# Supplementary material for: Rabies Vaccination and Public Health Insights in the Extended Arabian Gulf and Saudi Arabia: A Systematic Scoping Review
Source: Diseases. 2025 Apr 21;13(4):124. doi: 10.3390/diseases13040124 (PMC12025472; doi:10.3390/diseases13040124)
Supplement: Supplementary file 1 [file diseases-13-00124-s001.zip › Supplementary File S2.docx]

Table S1. Details of searching of each database.

| Database | Search strategy | Num of results |
| --- | --- | --- |
| Pubmed | ((Rabies Vaccines OR Vaccines, Rabies OR Rabies Vaccine OR Vaccine, Rabies OR Rabies Human Diploid Cell Vaccine OR Rabies Vaccine OR Vaccine, Rabies OR ((Rabies OR Hydrophobia OR Lyssa OR Lyssas OR Paralytic Rabies OR Encephalitic Rabies OR Rabies, Encephalitic OR Furious Rabies OR Furious Raby OR Rabies, Furious OR Raby, Furious OR rabies disease) AND (vaccine OR vaccines))) AND (Saudi Arabia OR Bahrain OR Iraq OR Kuwait OR Oman OR Qatar OR United Arab Emirates OR Southwest Asia OR Iran OR West Asia OR Western Asia OR persian gulf OR Arabian Gulf OR Baḥr Fāris OR Khalīj-e Fārs OR Gulf of Iran OR Gulf of Ajam OR The Gulf OR al-Khalīj al-ʻArabī OR Arab Gulf OR Saudi Arabian Peninsula OR The kingdom of Saudi Arabia OR middle east)) | 682 |
| Cochrane | ((Rabies Vaccines OR Vaccines, Rabies OR Rabies Vaccine OR Vaccine, Rabies OR Rabies Human Diploid Cell Vaccine OR Rabies Vaccine OR Vaccine, Rabies OR ((Rabies OR Hydrophobia OR Lyssa OR Lyssas OR Paralytic Rabies OR Encephalitic Rabies OR Rabies, Encephalitic OR Furious Rabies OR Furious Raby OR Rabies, Furious OR Raby, Furious OR rabies disease) AND (vaccine OR vaccines))) AND (Saudi Arabia OR Bahrain OR Iraq OR Kuwait OR Oman OR Qatar OR United Arab Emirates OR Southwest Asia OR Iran OR West Asia OR Western Asia OR persian gulf OR Arabian Gulf OR Baḥr Fāris OR Khalīj-e Fārs OR Gulf of Iran OR Gulf of Ajam OR The Gulf OR al-Khalīj al-ʻArabī OR Arab Gulf OR Saudi Arabian Peninsula OR The kingdom of Saudi Arabia OR middle east)) | 5 |
| Medline | ((Rabies Vaccines OR Vaccines, Rabies OR Rabies Vaccine OR Vaccine, Rabies OR Rabies Human Diploid Cell Vaccine OR Rabies Vaccine OR Vaccine, Rabies OR ((Rabies OR Hydrophobia OR Lyssa OR Lyssas OR Paralytic Rabies OR Encephalitic Rabies OR Rabies, Encephalitic OR Furious Rabies OR Furious Raby OR Rabies, Furious OR Raby, Furious OR rabies disease) AND (vaccine OR vaccines))) AND (Saudi Arabia OR Bahrain OR Iraq OR Kuwait OR Oman OR Qatar OR United Arab Emirates OR Southwest Asia OR Iran OR West Asia OR Western Asia OR persian gulf OR Arabian Gulf OR Baḥr Fāris OR Khalīj-e Fārs OR Gulf of Iran OR Gulf of Ajam OR The Gulf OR al-Khalīj al-ʻArabī OR Arab Gulf OR Saudi Arabian Peninsula OR The kingdom of Saudi Arabia OR middle east)) | 93 |
| Scopus | ((“Rabies Vaccines” OR “Vaccines, Rabies” OR “Rabies Vaccine” OR “Vaccine, Rabies” OR “Rabies Human Diploid Cell Vaccine” OR “Rabies Vaccine” OR “Vaccine, Rabies” OR ((Rabies OR Hydrophobia OR Lyssa OR Lyssas OR “Paralytic Rabies” OR “Encephalitic Rabies” OR “Rabies, Encephalitic” OR “Furious Rabies” OR “Furious Raby” OR “Rabies, Furious” OR “Raby, Furious” OR “rabies disease”) AND (vaccine OR vaccines))) AND (“Saudi Arabia” OR Bahrain OR Iraq OR Kuwait OR Oman OR Qatar OR “United Arab Emirates” OR “Southwest Asia” OR Iran OR “West Asia” OR “Western Asia” OR “persian gulf” OR “Arabian Gulf” OR “Baḥr Fāris” OR “Khalīj-e Fārs” OR “Gulf of Iran” OR “Gulf of Ajam” OR “The Gulf” OR “al-Khalīj al-ʻArabī” OR “Arab Gulf” OR “Saudi Arabian Peninsula” OR “The kingdom of Saudi Arabia” OR “middle east”)) | 147 |
| WoS | ((“Rabies Vaccines” OR “Vaccines, Rabies” OR “Rabies Vaccine” OR “Vaccine, Rabies” OR “Rabies Human Diploid Cell Vaccine” OR “Rabies Vaccine” OR “Vaccine, Rabies” OR ((Rabies OR hydrophobic OR lassa OR lysias OR “Paralytic Rabies” OR “Encephalitic Rabies” OR “Rabies, Encephalitic” OR “Furious Rabies” OR “Furious Raby” OR “Rabies, Furious” OR “Raby, Furious” OR “rabies disease”) AND (vaccine OR vaccines))) AND (“Saudi Arabia” OR Bahrain OR Iraq OR Kuwait OR Oman OR Qatar OR “United Arab Emirates” OR “Southwest Asia” OR Iran OR “West Asia” OR “Western Asia” OR “persian gulf” OR “Arabian Gulf” OR “Baḥr Fāris” OR “Khalīj-e Fārs” OR “Gulf of Iran” OR “Gulf of Ajam” OR “The Gulf” OR “al-Khalīj al-ʻArabī” OR “Arab Gulf” OR “Saudi Arabian Peninsula” OR “The kingdom of Saudi Arabia” OR “middle east”)) (All Fields) \| | 204 |

**Table S2: More details of the baseline characteristics of the included patients.**

| **Study ID** | **Season N (%)** | **Previous rabies vaccine N (%)** | **Number of bites N (%)** | **Injury status N (%)** |
| --- | --- | --- | --- | --- |
| Khoubfekr et al. 2024 (1) | NA | NA | One 1 (50)  Multiple 1 (50) | NA |
| Davarani et al. 2023 (2) | NA | NA | 1 205 (21.9)  2 541 (57.9)  3 113 (12.1)  >3 74 (7.9) | NA |
| Khazaei et al. 2023 (3) | Spring 70215 (26.96)  Summer 64492 (24.76)  Fall 63435 (24.35)  Winter 62328 (23.93) | NA | NA | NA |
| Yıldırım et al. 2022 (4) | Spring 150 (20.1)  Summer 235 (31.4)  Autumn 207 (27.7)  Winter 156 (20.9) | 107 (14.3) | NA | NA |
| Bay et al. 2021 (5) | NA | NA | NA | NA |
| Celiloglu et al. 2021 (6) | NA | 11 (1.2) | NA | NA |
| Oztoprak et al. 2021 (7) | NA | NA | Most of the cases 2324 (92.5) were injured from one site of their body. | NA |
| Porsuk et al. 2021 (8) | NA | NA | NA | NA |
| Amiri et al. 2020 (9) | NA | NA | NA | NA |
| Can et al. 2020 (10) | Winter 161 (23.3)  Spring 194 (28.1)  Summer 168 (24.3)  Autumn 168 (24.3) | NA | NA | Superficial 350 (50.7)  Deep 341 (49.3) |
| Janatolma Kan et al. (2020) (11) | Spring 1600 (28.5)  Summer 1495 (26.7)  Autumn 1329 (23.7)  Winter 1180 (21.1) | NA | NA | NA |
| Rahmanian et al. 2020 (1) | Spring 90 (24.00)  Summer 76 (20.30)  Autumn 102 (27.20)  Winter 107 (28.50) | NA | NA | NA |
| Rasooli et al. 2020 (12) | NA | NA | NA | NA |
| Sarbazi et al. 2020 (13) | NA | NA | NA | NA |
| Hamta et al. 2019 (14) | NA | NA | One 1311 (54.3)  Two 631 (26.13)  Three 255 (1p.56)  More than 3 196 (8.11) | NA |
| Kassiri et al. 2018 (15) | Spring 666 (26.7)  Summer 586 (23.5)  Autumn 627 (25.2)  Winter 614 (24.6) | NA | NA | NA |
| Khazaei et al. 2018 (16) | Spring 938 (64.78)  Summer 292 (20.17)  Autumn 112 (7.73)  Winter 106 (7.32) | NA | One 625 (43.28)  Two 510 (35.32)  Three 199 (13.78)  ≥Four 110 (7.62) | Deep 303 (20.93)  Superficial 1145 (79.07) |
| BabazadeH. et al. 2016 (17) | Summer 566 (32.8)  Wnter 322 (18.7%) | NA | NA | NA |
| Ramezankhani et al. 2016 (18) | NA | NA | NA | NA |
| Amiri et al. 2015 (19) | NA | 107 (6.04) | NA | NA |
| Riabi et al. 2015 (20) | NA | NA | NA | Superficial wounds 611 (99.2)  Deep wounds 5 (0.8%) |
| Poorolajal et al. 2015 (21) | Spring 6336 (27.87)  Summer 6307 (27.74)  Winter 4864 (21.39)  Autumn 5229 (23.00) | NA | NA | Surface 11683 (79.01)  Deep 3104 (20.99) |
| Farahtaj et al. 2014 (22) | NA | NA | Single 5 (31.25)  Multiple 11 (68.75) | NA |
| Karbeyaz et al. 2014 (23) | NA | NA | NA | NA |
| Seri et al. 2014 (24) | NA | Yes 51 (3.1)  No 1634 (96.9) | One 1443 (85.7)  More than one 242 (14.3) | NA |
| Charkazi et al. 2013 (25) | Spring 3792 (28.8)  Winter 3481 (26.5)  Summer 3189 (24.3)  Autumn 2680 (20.4) | NA | NA | Superficial 1533 (11.7)  Deep 43 (3)  Not clear 97 (7) |
| Taghvaii et al. 2013 (26) | Summer 3220 (23)  Winter 3283 (23.3) | NA | NA | Superficial 13576 (96.7)  Deep 461 (3.3) |
| Ghannad et al. 2012 (27) | NA | NA | NA | NA |
| Ansari et al. 2011 (28) | NA | NA | NA | NA |
| Bijari et al. 2011 (29) | Spring 439 (26.4)  Summer 441 (26.5)  Autumn 382 (23)  Winter 400 (24.1) | NA | 1 730 ,(43.9)  2 538 (32.4)  3 394 (23.7) | Superficial 460 (27.7)  Deep 1202 (72.3) |
| Najafi et al. 2009 (30) | NA | NA | NA | NA |
| Sheikholeslami et al. 2009 (31) | NA | NA | NA | Deep 231 (15)  Superficial 1079 (70)  Deep plus superficial injuries 139 (9)  The extent of the injury was unknown 93 (6) |
| Kilic et al. 2006 (32) | Summer 486 (31)  Winter  Fall 377 (24) | NA | NA | NA |
| Sengoz et al. 2006 (33) | NA | NA | NA | Deep wounds 1250 (17)  Superficial wounds 6010 (83) |
| Bizri et al. 2000 (34) | NA | NA | NA | NA |
| Tabbara et al. 1995 (35) | NA | NA | NA | Superficial 2 (100) |
|  |  |  |  |  |

References

1. Khoubfekr H, Jokar M, Rahmanian V, Blouch H, Shirzadi MR, Bashar RJAPJoTM. Fatal cases in pediatric patients after post-exposure prophylaxis for rabies: A report of two cases. 2024;17(1):39-42.

2. Davarani ER, Domari AA, Mahani AH, Samakkhah SA, Raesi R, Daneshi SJTOPHJ. Epidemiological characteristics, injuries, and rabies post-exposure prophylaxis among children in Kerman county, Iran during 2019-2021. 2023;16(1).

3. Khazaei S, Karami M, Veisani Y, Solgi M, Goodarzi SJBoE, Trauma. Epidemiology of animal bites and associated factors with delay in post-exposure prophylaxis; a cross-sectional study. 2018;6(3):239.

4. Yıldırım AA, Doğan A, Kurt C, Çetinkol Y. Evaluation of Our Rabies Prevention Practices: Is Our Approach Correct? Iran J Public Health. 2022;51(9):2128-34.

5. Bay V, Rezapour A, Jafari M, Maleki MR, Asl IMJJoAD. Healthcare utilization patterns and economic burden of animal bites: A cross-sectional study. 2021;10(4):142-6.

6. Celiloglu C, Ozdemir U, Tolunay O, Sucu A, Celik UJJ-JOTCOP, PAKISTAN S. Post-exposure rabies prophylaxis for children in Southern Turkey. 2021;31(10).

7. Oztoprak N, Berk H, Kizilates F. Preventable public health challenge: Rabies suspected exposure and prophylaxis practices in southwestern of Turkey. Journal of Infection and Public Health. 2021;14(2):221-6.

8. Porsuk AO, Cerit CJTJoIiDC. An increasing public health problem: Suspected rabies exposures. 2021;15(11):1694-700.

9. Amiri S, Maleki Z, Nikbakht H-A, Hassanipour S, Salehiniya H, Ghayour A-R, et al. Epidemiological Patterns of Animal Bites in the Najafabad, Center of Iran (2012–2017). 2020;86(1).

10. Can FK, Tekin E, Sezen S, Clutter PJJoEN. Assessment of rabies prophylaxis cases in an emergency service. 2020;46(6):907-13.

11. Janatolmakan M, Delpak M, Abdi A, Mohamadi S, Andayeshgar B, Khatony AJBph. Epidemiological study on animal bite cases referred to Haji Daii health Center in Kermanshah province, Iran during 2013–2017. 2020;20:1-8.

12. Rasooli A, Pourhossein B, Bashar R, Shirzadi MR, Amiri B, Kheiri EV, et al. Case Report: Investigating Possible Etiologies of Post-Exposure Prophylaxis Failure and Deaths From Rabies Infection.

13. Sarbazi E, Sarbazi M, Ghaffari-Fam S, Babazadeh T, Heidari S, Aghakarimi K, et al. Factors related to delay in initiating post-exposure prophylaxis for rabies prevention among animal bite victims: a cross-sectional study in Northwest of Iran. 2020;8(4):236.

14. Hamta A, Saghafipour A, Hosseinalipour SA, Rezaei F. Forecasting delay times in post-exposure prophylaxis to human animal bite injuries in Central Iran: A decision tree analysis. Vet World. 2019;12(7):965-71.

15. Kassiri H, Ebrahimi A, Lotfi MJAoCID. Animal bites: epidemiological considerations in the east of Ahvaz County, Southwestern Iran (2011-2013). 2018;13(5).

16. Khazaei S, Shirzadi MR, Amiri B, Pourmozafari J, Ayubi EJJoRiHS. Epidemiologic aspects of animal bite, rabies, and predictors of delay in post-exposure prophylaxis: A national registry-based study in Iran. 2023;23(2).

17. Babazadeh T, Nikbakhat HA, Daemi A, Yegane-Kasgari M, Ghaffari-Fam S, Banaye-Jeddi MJJoAd. Epidemiology of acute animal bite and the direct cost of rabies vaccination. 2016;5(6):488-92.

18. Ramezankhani R, Shirzadi MR, Ramezankhani A, POOR MJ. A comparative study on the adverse reactions of purified chick embryo cell vaccine (PCECV) and purified vero cell rabies vaccine (PVRV). 2016.

19. Mohtasham-Amiri Z, Pourmarzi D, Razi M. Epidemiology of dog bite, a potential source of rabies in Guilan, north of Iran. Asian Pacific Journal of Tropical Disease. 2015;5:S104-S8.

20. Riabi HRA, Ghorbannia R, Mazlum SB, Atarodi AJJoC, JCDR DR. A Three-year (2011–2013) Surveillance on Animal Bites and Victims Vaccination in the South of Khorasan-e-Razavi Province, Iran. 2015;9(12):LC01.

21. Poorolajal J, Babaee I, Yoosefi R, Farnoosh FJAoIm. Animal bite and deficiencies in rabies post-exposure prophylaxis in Tehran, Iran. 2015;18(12):0-.

22. Farahtaj F, Fayaz A, Howaizi N, Biglari P, Gholami AJTD. Human rabies in Iran. 2014;44(4):226-9.

23. Karbeyaz K, Ayranci UJJofs. A Forensic and Medical Evaluation of Dog Bites in a Province of W estern T urkey. 2014;59(2):505-9.

24. Sari T, Tulek N, Bulut C, Oral B, Ertem GTJTM, Disease I. Adverse events following rabies post-exposure prophylaxis: A comparative study of two different schedules and two vaccines. 2014;12(6):659-66.

25. Charkazi A, Behnampour N, Fathi M, Esmaeili A, Shahnazi H, Heshmati H. Epidemiology of animal bite in Aq Qala city, northen of Iran. J Educ Health Promot. 2013;2:13.

26. Taghvaii MRE, Seyednozadi SMJB, Journal P. An epidemiologic survey on animal bite cases referred to health centers in Mashhad during 2006 to 2009. 2015;6(2):301-6.

27. Ghannad MS, Roshanaei G, Rostampour F, Fallahi AJAoIm. An epidemiologic study of animal bites in Ilam Province, Iran. 2012;15(6):0-.

28. Ansari M, Shafiei M, Kordi RJAjosm. Dog bites among off-road cyclists: a report of two cases. 2012;3(1):60.

29. Bijari B, Sharifzade GR, Abbasi A, Salehi SJAoCID. Epidemiological survey of animal bites in east of Iran. 2011;6(2):90-2.

30. Najafi NJAoCID. Animal bites and rabies in northern Iran, 2001–2005. 2009;4(4).

31. Sheikholeslami N, Rezaeian M, Salem ZJE-EMHJ, 15 , 455-457,. Epidemiology of animal bites in Rafsanjan, southeast of Islamic Republic of Iran, 2003-05. 2009.

32. Kilic B, Unal B, Semin S, Konakci SKJIjoid. An important public health problem: rabies suspected bites and post-exposure prophylaxis in a health district in Turkey. 2006;10(3):248-54.

33. Sengoz G, Yasar KK, Karabela SN, Yildirim F, Vardarman FT, Nazlican OJJjoid. Evaluation of cases admitted to a center in Istanbul, Turkey in 2003 for rabies vaccination and three rabies cases followed up in the last 15 years. 2006;59(4):254-7.

34. Bizri A, Azar A, Salam N, Mokhbat JJE, Infection. Human rabies in Lebanon: lessons for control. 2000;125(1):175-9.

35. Tabbara KF, al-Omar O. Eyelid laceration sustained in an attack by a rabid desert fox. Am J Ophthalmol. 1995;119(5):651-2.
